# Supplementary material for: Mendelian randomization study of whole blood viscosity and cardiovascular diseases
Source: PLoS One. 2024 Apr 26;19(4):e0294095. doi: 10.1371/journal.pone.0294095 (PMC11051600; doi:10.1371/journal.pone.0294095)
Supplement: Attachment — Submitted filename: response_to_reviewer_PONE-D-23-33347.docx [file pone.0294095.s006.docx]

**Journal requirements:**

1. When submitting your revision, we need you to address these additional requirements.

Please ensure that your manuscript meets PLOS ONE's style requirements, including those for file naming. The PLOS ONE style templates can be found at

<https://journals.plos.org/plosone/s/file?id=wjVg/PLOSOne_formatting_sample_main_body.pdf> and 
<https://journals.plos.org/plosone/s/file?id=ba62/PLOSOne_formatting_sample_title_authors_affiliations.pdf>.

🡺 We have re-checked the journal’s style requirements and updated the manuscript as follows:

*In the author list part, we changed affiliation indication to number.

*In the affiliation part, we removed ZIP or postal codes.

*In the corresponding authorship part, we excluded physical addresses and left only email addresses.

*In the manuscript, we have changed the font size and headings to the PLOS ONE template.

*In the manuscript, we have changed the citation of figures as Fig x.

*In the manuscript, we moved figure captions and table directly after the paragraph in which they are first cited.

2. We note that you have stated that you will provide repository information for your data at acceptance. Should your manuscript be accepted for publication, we will hold it until you provide the relevant accession numbers or DOIs necessary to access your data. If you wish to make changes to your Data Availability statement, please describe these changes in your cover letter and we will update your Data Availability statement to reflect the information you provide.

🡺 The data presented here is available in the UK Biobank, as stated in the manuscript. No changes are needed for the Data Availability statement.

3. Please include captions for your Supporting Information files at the end of your manuscript, and update any in-text citations to match accordingly. Please see our Supporting Information guidelines for more information: <http://journals.plos.org/plosone/s/supporting-information>.

🡺 We have included captions for Supporting information files at the end of the manuscript and updated in-text citations to match accordingly.

**Reviewers' comments:**

**Reviewer's Responses to Questions**

**Comments to the Author**

1. Is the manuscript technically sound, and do the data support the conclusions?

The manuscript must describe a technically sound piece of scientific research with data that supports the conclusions. Experiments must have been conducted rigorously, with appropriate controls, replication, and sample sizes. The conclusions must be drawn appropriately based on the data presented.

Reviewer #1: Yes

Reviewer #2: No

🡺 We have modified manuscript to accommodate reviewers’ suggestions. (see below point-by-point responses)

2. Has the statistical analysis been performed appropriately and rigorously?

Reviewer #1: Yes

Reviewer #2: No

🡺 We have presented responses to accommodate reviewers’ comments. (see below point-by-point responses)

3. Have the authors made all data underlying the findings in their manuscript fully available?

The [PLOS Data policy](http://www.plosone.org/static/policies.action#sharing) requires authors to make all data underlying the findings described in their manuscript fully available without restriction, with rare exception (please refer to the Data Availability Statement in the manuscript PDF file). The data should be provided as part of the manuscript or its supporting information, or deposited to a public repository. For example, in addition to summary statistics, the data points behind means, medians and variance measures should be available. If there are restrictions on publicly sharing data—e.g. participant privacy or use of data from a third party—those must be specified.

Reviewer #1: Yes

Reviewer #2: Yes

4. Is the manuscript presented in an intelligible fashion and written in standard English?

PLOS ONE does not copyedit accepted manuscripts, so the language in submitted articles must be clear, correct, and unambiguous. Any typographical or grammatical errors should be corrected at revision, so please note any specific errors here.

Reviewer #1: Yes

Reviewer #2: Yes

**5. Review Comments to the Author**

Please use the space provided to explain your answers to the questions above. You may also include additional comments for the author, including concerns about dual publication, research ethics, or publication ethics. (Please upload your review as an attachment if it exceeds 20,000 characters)

Reviewer #1: The association between whole blood viscosity (WBV) and increased risk of cardiovascular disease (CVD) has been reported. In this study, the causal relationship between WBV and CVD was investigated.
I have a few suggestions;

1-" I recommend you to review the articles titled "The relationship between blood viscosity and acute arterial occlusion" and "Relationship between whole blood viscosity and lower extremity peripheral artery disease severity".

🡺 Thank you for sharing the articles; they significantly contribute to the manuscript, providing a deeper understanding and insight for the readers. We have appropriately referenced these articles in the introduction section (line 5, page 3, reference numbers 6 and 7).

Reviewer #2: This study investigated the associations between a PRS for whole blood viscosity and major cardiovascular event (MACE) in the UK Biobank through a split sample approach. The selection of genetic instrument from GWAS is rigorous, using an allele score (PRS in this case) to collectively represent the genetic instruments is also valid, but the statistical analysis - linear or logistic regression using PRS and outcome is not technically an instrumental variable analysis (and thus not a Mendelian randomization study).

Using individual level data, MR should be preformed using two-stage least squares regression (see the Burgess et al. 2016 paper below). Otherwise, the authors could use the split sample approach in two-sample MR setting: using subset1 and subset2 to estimate the IV-exposure and IV-outcome associations, and repeat the analysis in the reversed way, finally, meta-analyse the results. The current statistical analysis only estimates the ASSOCIATION between genetic liabilities towards high/low WBV and observed MACE outcomes.

Burgess, S., Dudbridge, F., and Thompson, S. G. (2016) Combining information on multiple instrumental variables in Mendelian randomization: comparison of allele score and summarized data methods. Statist. Med., 35: 1880–1906. doi: 10.1002/sim.6835.

🡺 Thank you for providing your valuable insight into the methodology presented in the manuscript and offering guidance on the effective analysis method. In response to the reviewer’s comments, we have implemented the split sample approach in two-sample MR setting. The method section and the result section in the manuscript have been revised accordingly.

We retitled the Genetic instrument subsection to the Mendelian randomization and introduced the concept of split sample approach used in the study at the beginning of the section (from line 16 to 19, page 5).

We screened genetic variants by querying them one by one, as an error was found when we queried groups of variants together. Consequently, we discovered additional variants with evidence of pleiotropy that were overlooked in the previous analysis. These newly identified variants were subsequently filtered out in the current analysis (from line 18 to 20, page 6).

We derived causal estimates of whole blood viscosity on outcomes using the inverse-variance weighted method, weighted median, and MR-Egger method. This was performed twice, separately using IV-exposure associations from subset 1 and IV-outcome associations from subset 2, and vice versa. A single estimate was then derived by combing the results from these two analyses (from line 21, page 6 to line2, page 7).

The resulting MR analysis still indicates insufficient evidence for a causal relationship between whole blood viscosity and the outcome. We have made corresponding modifications to the result section (from line 8 to 14), figure 1, and Supplementary Table 5 to present the updated MR results. We have removed the contents derived from the results of the previous association-based study

6. PLOS authors have the option to publish the peer review history of their article ([what does this mean?](https://journals.plos.org/plosone/s/editorial-and-peer-review-process#loc-peer-review-history)). If published, this will include your full peer review and any attached files.

If you choose “no”, your identity will remain anonymous but your review may still be made public.

**Do you want your identity to be public for this peer review?** For information about this choice, including consent withdrawal, please see our [Privacy Policy](https://www.plos.org/privacy-policy).

Reviewer #1: No

Reviewer #2: No
