# Supplementary material for: Mendelian randomization study of whole blood viscosity and cardiovascular diseases
Source: PLoS One. 2024 Apr 26;19(4):e0294095. doi: 10.1371/journal.pone.0294095 (PMC11051600; doi:10.1371/journal.pone.0294095)
Supplement: Attachment — Submitted filename: response_to_reviewer_PONE-D-23-33347.docx [file pone.0294095.s007.docx]

**Journal requirements:**

Please review your reference list to ensure that it is complete and correct. If you have cited papers that have been retracted, please include the rationale for doing so in the manuscript text, or remove these references and replace them with relevant current references. Any changes to the reference list should be mentioned in the rebuttal letter that accompanies your revised manuscript. If you need to cite a retracted article, indicate the article’s retracted status in the References list and also include a citation and full reference for the retraction notice.

🡺 Thank you for revieing the manuscript. We have checked the list of references and edited the list of authors in the reference “Relationship between diabetes mellitus and blood viscosity as measured by the digital microcapillary® system.”, from “Andrea V, Timan I, editors” to “Andrea V and Timan I”. The authors have confirmed that the manuscript doesn’t include papers that have been retracted and are happy to revise it if the journal could provide the list of papers that the issue arose.

**Reviewers' comments:**

**Reviewer's Responses to Questions**

**Comments to the Author**

1. If the authors have adequately addressed your comments raised in a previous round of review and you feel that this manuscript is now acceptable for publication, you may indicate that here to bypass the “Comments to the Author” section, enter your conflict of interest statement in the “Confidential to Editor” section, and submit your "Accept" recommendation.

Reviewer #3: All comments have been addressed

Reviewer #4: All comments have been addressed

2. Is the manuscript technically sound, and do the data support the conclusions?

The manuscript must describe a technically sound piece of scientific research with data that supports the conclusions. Experiments must have been conducted rigorously, with appropriate controls, replication, and sample sizes. The conclusions must be drawn appropriately based on the data presented.

Reviewer #3: Yes

Reviewer #4: (No Response)

3. Has the statistical analysis been performed appropriately and rigorously?

Reviewer #3: Yes

Reviewer #4: (No Response)

4. Have the authors made all data underlying the findings in their manuscript fully available?

The [PLOS Data policy](http://www.plosone.org/static/policies.action#sharing) requires authors to make all data underlying the findings described in their manuscript fully available without restriction, with rare exception (please refer to the Data Availability Statement in the manuscript PDF file). The data should be provided as part of the manuscript or its supporting information, or deposited to a public repository. For example, in addition to summary statistics, the data points behind means, medians and variance measures should be available. If there are restrictions on publicly sharing data—e.g. participant privacy or use of data from a third party—those must be specified.

Reviewer #3: Yes

Reviewer #4: (No Response)

5. Is the manuscript presented in an intelligible fashion and written in standard English?

PLOS ONE does not copyedit accepted manuscripts, so the language in submitted articles must be clear, correct, and unambiguous. Any typographical or grammatical errors should be corrected at revision, so please note any specific errors here.

Reviewer #3: Yes

Reviewer #4: (No Response)

6. Review Comments to the Author

Please use the space provided to explain your answers to the questions above. You may also include additional comments for the author, including concerns about dual publication, research ethics, or publication ethics. (Please upload your review as an attachment if it exceeds 20,000 characters)

Reviewer #3: I have reviewed the manuscript entitled 'Mendelian Randomization Study of Whole Blood Viscosity and Cardiovascular Diseases'.
The manuscript mentions the role of WBV in cardiovascular diseases. The thrombus presencen has been linked to WBV in patients admitted to outpatient clinic. Please mention this link in the introduction section citing 'Association of whole blood viscosity with thrombus presence in patients undergoing transoesophageal echocardiography'.

🡺 Thank you for reviewing the manuscript and sharing the article; it significantly contributes to the manuscript, providing a broader understanding and invaluable insight for the readers. We have appropriately referenced the article in the introduction section (line 5, page 3, reference number 8).

Reviewer #4: The authors’ responses have answered all earlier comments. I don't have any further comments on the current version.

🡺 Thank you for reviewing the manuscript and providing your invaluable guidance. Your comments have enhanced the manuscript, offering a deeper understanding and invaluable insights for the readers. We greatly appreciate it.

7. PLOS authors have the option to publish the peer review history of their article ([what does this mean?](https://journals.plos.org/plosone/s/editorial-and-peer-review-process#loc-peer-review-history)). If published, this will include your full peer review and any attached files.


If you choose “no”, your identity will remain anonymous but your review may still be made public.


**Do you want your identity to be public for this peer review?** For information about this choice, including consent withdrawal, please see our [Privacy Policy](https://www.plos.org/privacy-policy).

Reviewer #3: No

Reviewer #4: No
